# CNT/Graphite/SBS Conductive Fibers for Strain Sensing in Wearable Telerehabilitation Devices

**DOI:** 10.3390/s22030800

**Published:** 2022-01-21

**Authors:** Piotr Walter, Bartłomiej Podsiadły, Marcin Zych, Michał Kamiński, Andrzej Skalski, Tomasz Raczyński, Daniel Janczak, Małgorzata Jakubowska

**Affiliations:** 1 Faculty of Mechatronics, Institute of Metrology and Biomedical Engineering, Warsaw University of Technology, 02-525 Warsaw, Poland; bartlomiej.podsiadly.dokt@pw.edu.pl (B.P.); marcin.j.zych@gmail.com (M.Z.); m.kaminski1097@gmail.com (M.K.); andrzej.skalski@pw.edu.pl (A.S.); tomasz.raczynski.dokt@pw.edu.pl (T.R.); Daniel.janczak@pw.edu.pl (D.J.); malgorzata.jakubowska@pw.edu.pl (M.J.); 2 Centre for Advanced Materials and Technologies, Warsaw University of Technology, 02-822 Warsaw, Poland

**Keywords:** strain sensor, conductive polymer composite, conductive fiber, textile electronics

## Abstract

Rapid growth of personal electronics with concurrent research into telerehabilitation solutions discovers opportunities to redefine the future of orthopedic rehabilitation. After joint injury or operation, convalescence includes free active range of movement exercises, such as joints bending and straightening under medical supervision. Flexion detection through wearable textile sensors provides numerous potential benefits such as: (1) reduced cost; (2) continuous monitoring; (3) remote telerehabilitation; (4) gamification; and (5) detection of risk-inducing activities in daily routine. To address this issue, novel piezoresistive multi-walled carbon nanotubes/graphite/styrene–butadiene–styrene copolymer (CNT/Gr/SBS) fiber was developed. The extrusion process allowed adjustable diameter fiber production, while being a scalable, industrially adapted method of manufacturing textile electronics. Composite fibers were highly stretchable, withstanding strains up to 285%, and exhibited exceptional piezoresistive parameters with a gauge factor of 91.64 for 0–100% strain range and 2955 for the full scope. Considering the composite’s flexibility and sensitivity during a series of cyclic loading, it was concluded that developed Gr/CNT/SBS fibers were suitable for application in wearable piezoresistive sensors for telerehabilitation application.

## 1. Introduction

The recent prevalence of personal electronics in everyday usage holds a great premise towards the rapid development of rehabilitation-assisting devices. Investigation of new and emerging rehabilitation modalities has gained even more significance during the COVID-19 pandemic, as telemedicine and telerehabilitation have been widely adopted. The opportunity to remotely consult patients, verify rehabilitation progress, and adjust exercises accordingly is not the only advantage. Monitoring orthopedic rehabilitation through personal electronics devices allows the acquisition of motor metrics and biomarkers outside the doctor’s office. It improves the reliability of the assessment because (1) patients can be monitored over an extended period; (2) individual’s motor performance is not influenced by ongoing clinical examination, which can distort typical motor patterns and spatiotemporal parameters. Numerous research has reported non-inferiority of in-home telerehabilitation in comparison with face-to-face rehabilitation [1,2]. Moreover, wearable medical devices offer the opportunity to gamify the orthopedic rehabilitation process to motivate participants, which has been proven effective in several fields of disabilities [3,4].

After joint injury or operation, convalescence includes free active range of movement exercises, such as bending and straightening of the joints. Instead of continuous professional observation, whenever possible, the patient could perform the exercises independently with feedback from the wearable flexion sensor. This could greatly improve the rehabilitation process, reducing the necessary time of medical supervision. Venkataraman et al. described “although formal gait analysis using motion-capture systems is the gold standard for evaluating gait and mobility performance, such analysis is expensive and requires a specialized laboratory setup that may not be feasible or practicable for clinical or home settings” [5]. Low-cost, wearable sensors would also enable continuous monitoring of the patient’s everyday activities to identify risk-inducing movements and prevent future health complications.

To detect joint flexion with a wearable sensor, flexible strain gauges are considered to be the most promising solution. Conventional metal strain gauges and extensometers fail to meet the requirements posed by capturing the garment elongation associated with joint flexion. Moreover, their gauge factor at ~2% and 5% of maximal strain [6] are insufficient for wearable sensor applications. Textile strain sensors must be lightweight, flexible, stretchable, strictly integrated with the garment structure, and withstand strains up to 55% [7]. Due to these requirements, conventional electrical solutions cannot be adopted to textile strain sensors. Apart from the flexibility aspect, there are challenges regarding unconventional substrate adhesion to the textile, thermal expansion compatibility, resistance to washing, environmental resistance, and stability of electrical parameters over time.

To meet the requirements posed by flexible, stretchable textronic sensors, substantial research has been devoted to the development of elastic conductive polymers composites (elastic CPCs). The vast majority of these solutions are composites based on conductive particles immobilized in an elastomer matrix. The conductive phase of the CPC may constitute of metal particles (gold [8,9], platinum [10], silver [11,12,13,14], copper [15,16], and nickel [17,18]), metal oxides (ZnO [19,20], Fe_3_O_4_ [21,22], and RuO_2_ [23]) carbon nanostructures (carbon black [24,25,26], graphite [27], graphene [28,29,30,31,32], and carbon nanotubes [32,33,34,35,36,37]) and their oxides (reduced graphene oxide [38,39] and graphene oxide [40]). The elastomer matrixes most commonly used are: poly (dimethylsiloxane) (PDMS) [9,20,28,31,35,41], TPU [33,38,42,43,44,45], PU [46,47,48], SBS [49,50], and Ecoflex [8,25,51,52].

Although CPCs outperform conventional electric materials in terms of mechanical properties, their usage in textile electronics poses two significant challenges—manufacturing process and stabilizing their electrical properties, especially under strains. Numerous CPC solutions have been reported, yet most manufacturing methods fail to scale into electronics production. The majority of these solutions are manufactured as separate, fully-functional structures, and only at the end are they coupled with the substrate using gluing [53,54,55], stitching [56], or fastening with adhesive tape [14,54,57,58,59]. These methods are not repeatable or/and are hand labor-intensive and therefore not scalable for high-volume production. However, the application of screen printing and heat transfer printing has been reported [60,61,62]. These manufacturing methods enable large-format, scalable, time-efficient production of sensors firmly embedded in the textile fibers of the substrate.

Although screen-printing techniques offer numerous advantages, their usage in flexible strain sensors is limited by several factors. Firstly, screen-printing requires a relatively uniform, planar surface of the substrate. It is particularly challenging considering various forms of orthoses and fiber thickness of the bands stabilizing the joints. Secondly, due to low layer thickness, screen-printed strain gauges often require a higher print area over the textile substrate in comparison with fibers. The polymer layer impairs uniformity of the textile elasticity, which can significantly alter the stretching of the fabric, restrict the user’s movement, and result in additional creasing of the fabric. In response to mentioned requirements, substantial research has been conducted towards the development of conductive fibers in fiber-based electronics that are expected to be lightweight, long-lasting, flexible, and conformable [7].

Therefore, we present a novel CPC piezoresistive fiber intended to meet the requirements of textile, fiber-based electronics and suitable for strain sensing in telerehabilitation application. Our composite is based on the elastomer–styrene–butadiene–styrene copolymer (SBS), with the addition of a carbon conductive phase. Numerous proportions of graphite (Gr) and multi-walled carbon nanotubes (CNT, MWCNT) and SBS elastomer have been tested. Out of 14 compositions, 4 with superior conductivity were selected and further characterized with strain testing, piezoresistive testing, and cyclic loading. Fibers with diameters of 1 mm, 0.5 mm, and 0.2 mm have been successfully manufactured from prepared granulate. The employed method of hot extrusion allows scalable, bulk production of continuous fibers with adjustable diameter, determined by an interchangeable nozzle. The developed composite fibers exhibit exceptional piezoresistive parameters with a gauge factor up to 2955 and maximal strain over 200%. With their high flexibility and small diameter, the fibers can be easily integrated as textronics strain gauges by knitting directly onto the garment.

## 2. Experimental

### 2.1. Fiber Preparation

Utilized composite substrates consisting of styrene–butadiene–styrene (SBS) triblock copolymer Europrene SOL T 166 (Versalis, San Donato Milanese, Italy), graphite (Gr) powder MG1596 (Sinograf SA, Toruń, Poland), multiwall carbon nanotubes (CNT) NC7000 (Nanocyl SA, Sambreville, Belgium), and chloroform (Merck KGaA, Darmstadt, Germany).

SBS was prepared by mixing copolymer granulate with chloroform (50 wt%) and subjected to ultrasound sonification until a homogenous solution was achieved. Similarly, CNT agglomerates and graphite were dispersed within separate chloroform solutions and 30′ of sonification. Carbon suspensions were mixed with SBS solution so that CNT to Gr to SBS weight ratios would correspond to the target filament’s yield composition (Table 1), as solvents are evaporated in the following steps. Solutions were thoroughly mixed using an MS7-H550-Pro magnetic stirrer for two hours to ensure uniform distribution of the carbon particles and initial evaporation of the solvent. Then, the solution was mixed by hand, poured onto a large area container, followed by drying at 50 °C for 24 h to evaporate the remaining chloroform. The composite cast was fragmented with pliers into ~1 cm chunks and poured into the hopper of the extruder. A single screw extruder with dual heating zones (set to 150 °C) was incorporated to manufacture continuous CPC fibers with diameters of 0.2 mm, 0.5 mm, and 1 mm corresponding to the interchangeable extruder’s nozzle diameter.

### 2.2. Carbon Content Selection

To establish a filament’s yield target carbon content, numerous filaments of various CNT/SBS, Gr/SBS, and CNT/Gr/SBS ratios were prepared (Table 1). The preliminary composition selection was carried out based on the electrical conductivity requirement. For application on knee-stabilizing bands with joint flexion detection, 10 cm of the flexible filament was assumed as the target length. To ensure an appropriate resistance measurement for the portable and wearable electrical module, a subjective maximum resistance value of 5·10^6^ Ω was established for the stretched fibers. A 50-fold increase in initial resistance for 50% elongation was assumed through preliminary testing. Therefore, for 10 cm in length filaments, a maximum initial resistance value of 1·10^5^ Ω was desired. Assuming 0.5 mm filament diameter, the calculated value of 5 S·m^−1^ was the target minimal conductivity for composite selection. Fibers with graphite content above 50% and fibers with over 10% CNTs failed to extrude due to the rheology of the melted mixture. FGr, FCNT, FM1, and FM2 fibers of 0.5 mm diameter were chosen for further characterization.

### 2.3. SBS Fiber Characterization

Static tensile tests and fibers’ critical strain were investigated with the Cometech QC-506M2 tensile testing machine (Cometech Testing Machines Co., Ltd., Taichung City, Taiwan). Fibers were characterized at 50 mm in-between jaws length, and custom soft jaws were employed to reduce the stresses resulting from the specimen fixing. The critical strain was calculated as maximal elongation at the breaking point relative to the initial sample length. Preliminary conductivity qualification of the fibers was performed by 2-point resistance measurement using Fluke 177 multimeter (Fluke Corporation, Everett, WA, USA). Further conductivity assessment was established with 4-wire resistance probing with Keysight 34461A multimeter (Keysight Technologies, Santa Rosa, CA, USA).

The influence of stretching of the samples on its electrical resistance was investigated on a self-made device. The device allows mounting of the specimen in copper clamps, while one is fixed and the other can be moved by a stepper motor with a screw gear. Current elongation and electrical resistance are recorded in 25 ms intervals and transferred to the personal computer. The measurement range of resistance is from 2 Ω to 50 MΩ with an accuracy of 2%; the linear range of the movable clamp is 200 mm. Three samples with a diameter of 0.5 mm were selected to examine the given composite. Linear stretching was carried out at a speed of 0.15 mm·s^−1^, while resistance was probed at 39 Hz frequency. The samples were mounted in the clamps with a 10 mm distance between each clamp. Cyclic stretching was carried out for the FM1 fiber at a speed of 0.5 mm·s^−^ for preliminary cyclic loading, addressing dynamics of resistivity drop at the unloaded state, and 8 min of idle time was set after every 10% stretch–release cycle. For the 500-cycle testing, the same parameters and mounting were used, but at 20% strain cycles and no idle time between cycles. For clarity of the graphical representation, only one point indicated resistance in the stretched/released cycle, which was the maximum value measured in a 0.5 s span after the encoder recorded the target position.

## 3. Results and Discussion

**Figure 1:**
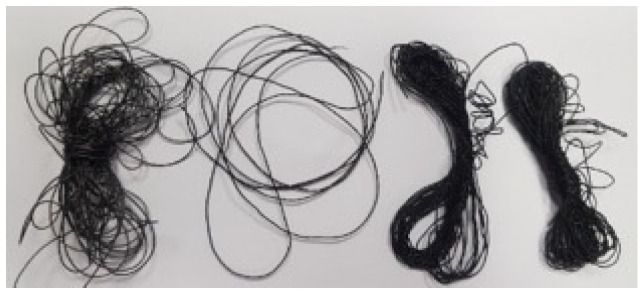
Coils of extruded composite fibers. From left to right, FGr (Gr/SBS), FCNT (CNT/SBS), FM1 (CNT/Gr/SBS), FM2 (CNT/Gr/SBS) are shown, respectively.

Extruded carbon-SBS fibers were highly elastic, which allowed for their storage in coils. Composites containing carbon nanotubes are matte black in color, while graphite/SBS fibers exhibit a gray appearance with metallic gloss (Figure 1).

For application as strain sensors in wearable textronic systems, fibers should withstand the associated elongation of the garment structure during joint flexion. Human skin during limb movements undergoes stretching over 100%, with local extensions up to 400%, as reported by on-skin sensor measurements [13]. However, garment textiles typically experience elongation under 10% since they do not exhibit mechanical compliance with the body and allow for movement of the garment relative to the skin surface. Most common yarns like cotton, wool, silk, bamboo, viscose, polyester, or polyamide fibers exhibit elongation at break in the range of 7–41% [63,64,65].

**Figure 2:**
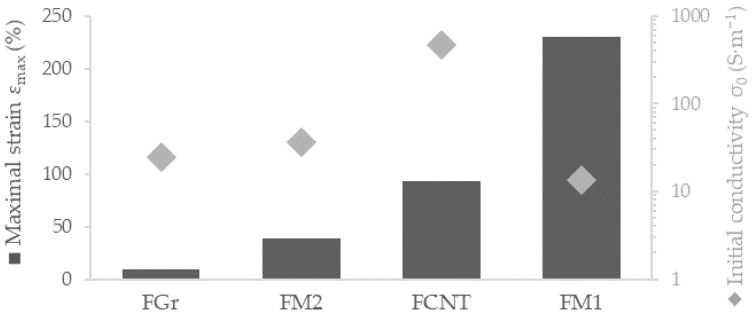
Breaking strain for different carbon/SBS composite 0.5 mm fibers with the assessment of the conductivity of the unstrained fiber. FGr is Gr/SBS filament; FCNT is CNT/SBS composition; and FM1 and FM2 are mixes of SBS, graphite, and CNT in different ratios.

However, for capturing joint flexion, it is necessary to employ a tightly-fitted garment made from highly elastic synthetic fibers such as nylon or elastane to ensure textile elongations compliance with skin movement. Generally for wearable sensors, the strain associated with joint flexion is under 50% [66,67] with reported strains varying from ~40% for finger bending [68,69,70,71,72], 23–45% for wrist movement [41,66,68,69,72], 35–63% for elbow flexion [68,69,72], and 30–40% for bending of the knee [68,72,73]. Values measured with knee flexion sensors are congruent with the motion capture analysis conducted by Wessendorf et al., which provides a value of 44.6% as maximum skin strain (in any direction) associated with the knee joint for full flexion and the extension cycle [74]. Wu et al. reported 40% maximal strain of the textronic system embedded onto the kneecap area of tight-fitting exercise pants [73]. This value was used as a base strain benchmark of our fibers, as they are intended for motion-capturing telerehabilitation devices. Therefore, with the employment of a universal testing machine, the critical strain of various compositions was established for fibers 0.5 mm in diameter (Figure 2).

**Figure 3:**
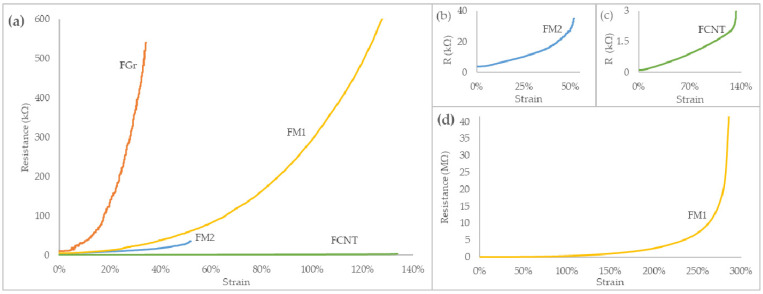
Piezoresistive characteristic of the fibers. (**a**) Resistance curve under strain for 0.5 mm fibers FGr, FCNT, FM1, and FM2; (**b**–**d**) R/ε plots for individual FM2, FCNT, and FM1 fibers.

Only FCNT and FM1 have sufficient elasticity to be applicable in wearable strain sensors (>40%), with FM1 exhibiting outstanding 230% elongation at break and FCNT breaking at 92% strain. All the fibers display elastic behavior while maintaining adequate conductivity at an unstrained state; therefore, piezoresistive testing was carried out (Figure 3) to establish linearity and sensitivity.

Gr/SBS filament FGr proved to have excellent sensitivity to stretching; however, its usage in strain sensors is limited due to its low breaking point. Fiber based on carbon nanotubes (FCNT) exhibits excellent linearity and strain working range; its gauge factor, however, is orders of magnitude lower than that of other filaments. As shown in the snippets of Figure 3, fibers FCNT and FM2 exhibit highly linear piezoresistive characteristics within the first 80% of their operating range. On the other hand, *R*/*ε* curves of FGr and FM1 are non-linear with a quasi-exponential course. Notably, of the two CNT/Gr/SBS filaments, FM1 displays an exceptional strain range with superior resistance change. Therefore, FM1 was chosen as the most promising option for high-sensitivity, long-range strain sensing.

The presented piezoresistive curves ended with the breaking of the samples. It was prevalent that all the fibers exhibited higher maximal strain in piezoresistive tests compared with tensile testing. The trend was too evident to be explained by the dispersion of measurements. It is believed that the tensile strength of the fiber could be described by the weakest link theory, which assumes Weibull distribution of the flaws in the material, therefore providing fiber fracture probability as a function of the length of the specimen [75,76]:

(1) $$F\left(\sigma_{t}\right)=1-\exp\left[-\frac{l}{l_{0}}{\left(\frac{\sigma_{t}}{\beta }\right)}^{\alpha }\right]$$

where $\sigma_{t}$ stands for normalized tensile strength; l is the length of the specimen; and $l_{0}$ stands for reference gauge length, while *α* and *β* are parameters for shape and scale of the specimen. Since the initial Weibel’s description of the statistical theory of damage in materials in 1939 [77], numerous models have been developed to describe the mechanical strength of fibers [78,79], fiber-reinforced composites [80], and particle-filled elastomers [81] with very notable Payne and Mullins effect [82]. Nevertheless, these models indicate a strong correlation between the length of the gauge and the decrease in the specimen’s strength. For tensile testing, the 50 mm gauge length was chosen, as it is the prevalent gauge length for tensile testing. For piezoresistive tests, shorter 10 mm samples were used, as a consequence of 2 Ω to 50 MΩ resistance measuring range. Therefore, we believe that the reason for higher elongation at break in piezoresistive characterization is an effect of shorter gauge length and is explained by the distribution of weak points throughout the fiber length, as indicated by cited models.

For piezoresistive strain gauges, one of the most critical parameters is the gauge factor (*GF*), defined as the ratio of the relative change in resistance (Δ*R*/*R*_0_) to the engineering strain of the specimen (*ε*):

(2) $$GF=\frac{\Delta R/R_{0}}{\varepsilon }=\frac{\Delta R/R_{0}}{\Delta l/l_{0}}=\frac{\frac{R-R_{0}}{R_{0}}}{\frac{l-l_{0}}{l_{0}}}$$

where $l_{0}$ is the initial, unstrained length of the specimen, $R_{0}$ its initial resistivity; l is the total length of the strained composite, with R as its measured resistivity. For fibers FGr, FCNT, FM1, and FM2, the gauge factor was calculated in various strain ranges, as presented in Table 2.

**Figure 4:**
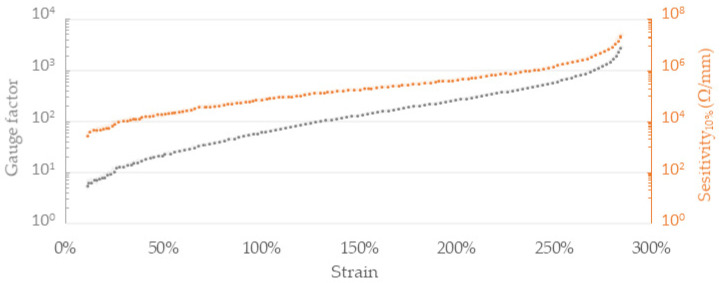
Gauge factor (GF) and sensitivity (S_10%_) calculated as a function of strain for 10 mm FM1 fiber.

As shown in Figure 3 and Table 2, piezoresistive behavior is manifested stronger in higher strain ranges. Considering the stretchability of FM1 fiber, fibers could be stretched prior to integration with the textile. If a particular application requires only 50% strain, then instead of 0–50% working range, the fiber could be working in 150–200% of its range to increase the piezoresistive response. Therefore, the sensitivity *S*_10%_ was calculated based on the resistance change over 1 mm of elongation, equivalent to 10% strain in 10 mm initial fiber length:

(3) $$S_{10\%}\left(\varepsilon \right)=\frac{R_{\varepsilon }-R_{\varepsilon -10\%}}{l_{10\%}}$$

where $R_{\varepsilon }$ is the resistance of the fiber under given strain ε, $R_{\varepsilon -10\%}$ is the resistance measured at a 10 percentage points lower strain and length of the specimen, and $l_{10\%}$ is an equivalent of 10% initial gauge length. A substantial increase in the $S_{10\%}$ and the gauge factor in relation to strain was observed (Figure 4), which is consistent with other reports of piezoresistive composite materials.

**Figure 5:**
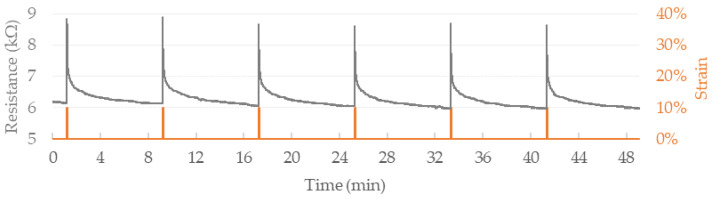
Cyclic strain testing of FM1 composite fiber. Strains of 10% were separated by 8 min of rest time to observe repeatability and dynamics of resistivity drop at unloaded state.

Noticeable sensitivity increase is observed in higher working ranges. For elongation between 0% and 10%, FM1 composite exhibits sensitivity of 2.7 kΩ/mm and the value increases to 24.4 MΩ/mm within 275–285%. Similarly, gauge factor increases from 5 at 10% to 2955 at 285%. The strain sensor in wearable textile application undergoes repeatable straining over movements of the body. To evaluate piezoresistive behavior under recurrent straining, cyclic testing of FM1 filament was conducted. Fibers of an initial length of 10 mm were strained by 10% at a speed of 0.5 mm·s^−1^ followed by an immediate return to the starting position at an equal pace. Multiple initial strains exhibited significantly higher resistance response than subsequent cycles; hence cycles 12–17 were chosen to represent the fiber’s performance under repetitive loading (Figure 5). A period of 1s of stretching and 1s of unstraining was followed by 8 min of rest time to ensure a return close to the baseline resistance—the extended idle time allowed to observe repeatability and dynamics of resistivity decrease after releasing the fiber.

**Figure 6:**
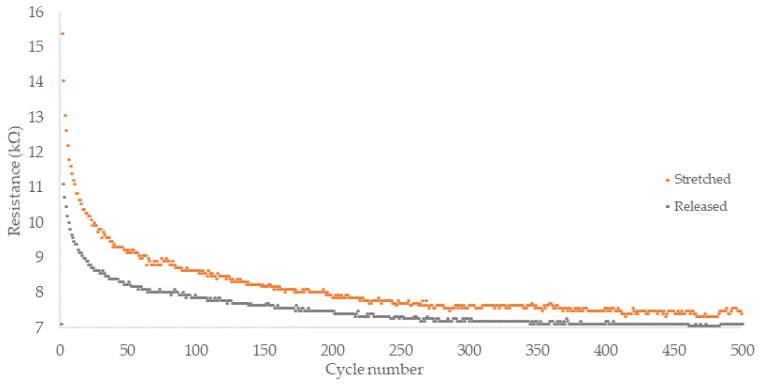
Cyclic strain testing of FM1 composite fiber. Strains of 20% were carried out by 2 s of stretching followed by immediate 2 s of releasing.

Novel CNT/Gr/SBS fiber exhibited satisfactory, repeatable response, however, a slight decrease in the resistance response can be observed for subsequent cycles. Conducted tests showed significant time (~5–8 min) needed for total resistance decrease (within ±2%), but only 5–9 s was necessary for 20% resistance decrease relative to peak value. Further cyclic testing was carried out to assess applicability for higher frequency (0.25 Hz) strain sensing and examine the manufactured fibers’ durability (Figure 6).

## 4. Conclusions

The extrusion process proved to be a suitable method for producing piezoresistive composite fibers. It provides a number of advantages: it is scalable, ideal for bulk manufacturing; allows for simple fiber diameter adjustment via an interchangeable nozzle; and produces continuous fibers with no limitations in length since the extruder’s hopper can be refilled during operation.

**Figure 7:**
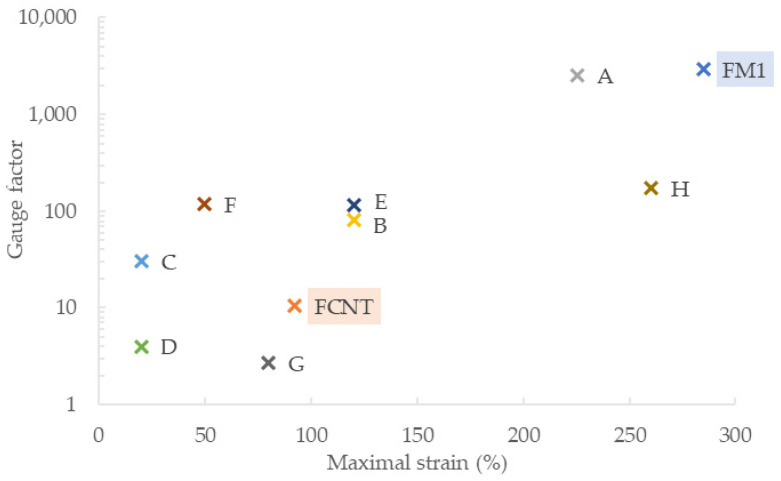
Comparison with previous works on piezoresistive composites based on carbon fillers and polymers from the styrene-butadiene family. Corresponding notation can be found in Table 3.

Fourteen CNT/SBS, Gr/SBS, and CNT/Gr/SBS composites were manufactured, consisting of various filler ratios. Each composite was subjected to an extrusion process with nozzles of 1 mm, 0.5 mm, and 0.2 mm in diameter. Out of 42 combinations, four 0.5 mm fibers were selected for further investigation—FCNT, FGr, FM1, and FMTensile tests revealed that only FCNT and FM1 withstand >40% of strains necessary for wearable strain sensor applications. Comparison with previous works on piezoresistive composites, based on polymers from the styrene–butadiene family and various carbon fillers, are presented in Figure 7 and Table 3.

Formerly reported CNT/SBS piezoresistive fibers utilizing the extrusion process had a gauge factor of 30 and a maximum strain of up to 20% [84]. Our FCNT composite exhibits a superior working range with the maximal strain of 92% and 134% for fiber lengths of 50 mm and 10 mm, respectively. The calculated gauge factor for the 0–100% range was 10.37 and 16.7 for the whole range until fiber rupture.

The proposed composite ratio of 10 wt% to 5 wt% to 85 wt% for CNT, Gr, and SBS respectively proved to yield even better results. Fiber FM1 consisting of the CNT/Gr/SBS composite exhibited a gauge factor of 60.55 for 100% strain range, 534.7 for 200%, and 2955 for the whole range up to rupture (285%). The presented CNT/Gr/SBS fiber exhibits superior strain range and gauge factor, compared with other reported works with composites based on poly (styrene–butadiene–styrene) elastomers. Notably, calculating GF near the fiber’s breaking point usually yields higher values, which are not indicative of working range sensitivity as achieving such strains is not repeatable due to significant fatigue. Lowering conductive filler content near the percolation threshold can also inflate gauge factor values, while introducing severe unrepeatability and irreproducibility. The developed CNT/Gr/SBS fiber has conductive filler content relatively distant from the percolation threshold, exhibiting satisfactory conductivity (13.6 ± 0.7 S·m^−1^) repeatably throughout over 5 m of extruded filament. Measuring fibers’ elongation at break showed the evident influence of the gauge length on the measurement outcome—230% for 50 mm length of FM1 and 285% for 10 mm. This correlation is supported by theoretical models in the literature devoted to conventional materials yet is frequently not considered in the piezoresistive composite reports.

Conducted testing revealed significant time (~5–8 min) necessary for the resistance value to return to the baseline. To assess applicability for the on-body telerehabilitation devices, cyclic strains at 0.25 Hz frequency were performed on the FM1 fiber. Throughout all 500 cycles, the dynamic of the resistance changes was sufficient, leaving the strain response clearly distinguishable from adjacent release values. While relative resistance change was apparent within the cycle, the absolute resistance value for the same 20% strain decreased significantly throughout the series, especially in the initial cycles. For application in wearable sensors, the electric signal from piezoresistive fiber has to be digitally analyzed to provide the current strain value accurately.

Combining carbon nanotubes and graphite in the SBS composite induces several advantages over CNT/SBS or graphite/SBS composites as summarized below:

Carbon nanotubes exhibit non-linear current/voltage characteristics; graphite, on the other hand, is highly linear within low electric fields—as observed in the literature [90,91] and our experimental data for Gr/SBS and CNT/SBS fibers. Combining CNT and graphite in the composite results in a nearly linear characteristic, while maintaining electromechanical advantages of carbon nanotubes content. Linearity of the U/I curve is desirable in sensor applications, as it results in a constant resistance response with respect to the applied probing voltage.

Composites with conductive particles of high aspect ratio, such as carbon nanotubes, tend to have significantly lower gauge factors than materials filled with low aspect ratio particles, such as graphite [24,43,92]. The addition of the graphite to the CNT/SBS composition greatly increased the sensitivity of the manufactured fibers at a similar initial conductivity baseline.

Carbon nanotubes are far more potent than graphite at improving carbon/SBS composite conductivity. Introducing CNT allows for lower wt% total carbon content at the same conductivity, which further enhances mechanical properties—stretchability and fatigue strength.

Reported methodology of preparing carbon nanotubes/graphite/poly (styrene–butadiene–styrene) composite fibers proved to attain excellent mechanical and piezoresistive behavior while maintaining a scalable, industrially adapted manufacturing process.

## Figures and Tables

**Table 1:** Conductive SBS fiber compositions of various CNT and Gr filler loading. Fibers with diameters of 0.2 mm, 0.5 mm, and 1 mm were extruded and assessed based on electrical conductivity.

Fiber Notation	Carbon Filler Loading		Conductivity Qualification		
	CNT wt%	Gr wt%	ø0.2 mm	ø0.5 mm	ø1 mm
-	-	10	—	—	—
-	-	20	—	—	—
-	-	30	—	—	—
-	-	40	—	—	—
-	-	45	—	—	—
FGr	-	50	+	+	—
-	2	-	—	—	—
-	5	-	—	—	—
FCNT	10	-	N/E	+	+
-	2	2	—	—	—
-	5	2	—	—	—
-	5	5	—	—	—
FM1	5	10	+	+	+
FM2	5	15	+	+	+

“+”—fiber conductivity exceeding 5 S m^−1^; “—”—fiber conductivity below 5 S m^−1^; N/E—fiber not extruded due to rheological performance.

**Table 2:** Gauge factor (GF) of the various composite filaments within several strain ranges. The last row describes the gauge factor of each filament for maximal strain as measured by piezoresistive testing (Figure 3).

Strain Range	FGr	FCNT	FM1	FM2
0–10%	25.05	3.88	5.34	4.94
0–20%	59.94	5.70	7.59	6.18
0–50%	-	7.90	20.75	12.71
0–100%	-	10.37	60.55	-
0–200%	-	-	524.7	-
0–max (*ε*_max_)	151.5 (34%)	16.70 (134%)	2955 (285%)	15.44 (52%)

**Table 3:** The composition of reported in the literature piezoresistive composites with carbon fillers and polymer matrices from the styrene-butadiene family.

Notation in Figure 7	Composite	Reference
FM1	CNT/Gr/SBS	This work
FCNT	CNT/SBS	This work
A	FLG/SBS	[83]
B	SBR/NR/Gr	[34]
C	CNT/SBS	[84]
D	CNT/SBS	[85]
E	GO/SEBS	[86]
F	CNT/SBS	[87]
G	CNT/SEBS	[88]
H	CNT/SBS	[89]

FLG—few-layer graphene; NR—natural rubber; SBR—styrene-butadiene rubber; SEBS—styrene-ethylene-butylene-styrene copolymer.

## Data Availability

All the data is available within the manuscript.
